# Effects of exercise-based pulmonary rehabilitation on adults with asthma: a systematic review and meta-analysis

**DOI:** 10.1186/s12931-021-01627-w

**Published:** 2021-01-30

**Authors:** Zhenzhen Feng, Jiajia Wang, Yang Xie, Jiansheng Li

**Affiliations:** 1 Dongzhimen Hospital, Beijing University of Chinese Medicine, Beijing, 100700 China; 2 Co-Construction Collaborative Innovation Center for Chinese Medicine and Respiratory Diseases By Henan & Education Ministry of P.R. China, Henan University of Chinese Medicine, 156 Jinshui East Road, Zhengzhou, 450046 China; 3 Henan Key Laboratory of Chinese Medicine for Respiratory Disease, Henan University of Chinese Medicine, Zhengzhou, 450046 China; 4 Department of Respiratory Diseases, The First Affiliated Hospital of Henan University of Chinese Medicine, Zhengzhou, 450000 China

**Keywords:** Pulmonary rehabilitation, Asthma, Systematic review, Meta-analysis

## Abstract

**Background:**

Pulmonary rehabilitation (PR) has been proposed as an effective method for many respiratory diseases. However, the effects of exercise-based PR on asthma are currently inconclusive. This review aimed to investigate the effects of exercise-based PR on adults with asthma.

**Methods:**

The PubMed, Embase, Cochrane Library, Web of Science, International Clinical Trials Registry Platform and ClinicalTrials.gov databases were searched from inception to 31 July 2019 without language restriction. Randomized controlled trials (RCTs) investigating the effects of exercise-based PR on adults with asthma were included. Study selection, data extraction and risk of bias assessment were performed by two investigators independently. Meta-analysis was conducted by RevMan software (version 5.3). Evidence quality was rated by the Grading of Recommendations, Assessment, Development and Evaluation (GRADE) system.

**Results:**

Ten literatures from nine studies (n = 418 patients) were identified. Asthma quality of life questionnaire total scores (MD = 0.39, 95% CI: 0.02 to 0.76) improved significantly in the experimental group compared to control group, including activity domain scores (MD = 0.58, 95% CI: 0.21 to 0.94), symptom domain scores (MD = 0.52, 95% CI: 0.19 to 0.85), emotion domain scores (MD = 0.53, 95% CI: − 0.03 to 1.09) and environment domain scores (MD = 0.56, 95% CI: 0.00 to 1.11). Both the 6-min walk distance (MD = 34.09, 95% CI: 2.51 to 65.66) and maximum oxygen uptake (MD = 4.45, 95% CI: 3.32 to 5.58) significantly improved. However, improvements in asthma control questionnaire scores (MD = − 0.25, 95% CI: − 0.51 to 0.02) and asthma symptom-free days (MD = 3.35, 95% CI: − 0.21 to 6.90) were not significant. Moreover, there was no significant improvement (MD = 0.10, 95% CI: − 0.08 to 0.29) in forced expiratory volume in 1 s. Nonetheless, improvements in forced vital capacity (MD = 0.23, 95% CI: 0.08 to 0.38) and peak expiratory flow (MD = 0.39, 95% CI: 0.21 to 0.57) were significant.

**Conclusions:**

Exercise-based PR may improve quality of life, exercise tolerance and some aspects of pulmonary function in adults with asthma and can be considered a supplementary therapy. RCTs of high quality and large sample sizes are required.

*Clinical trial registration:* The review was registered with PROSPERO (The website is https://www.crd.york.ac.uk/prospero/, and the ID is CRD42019147107).

## Background

Asthma, characterized by variable symptoms of wheezing, shortness of breath, chest tightness and/or cough, and variable expiratory airflow limitation, affects 1–18% of the population in different countries [1]. From 1990 to 2015, the prevalence of asthma increased by 12.6%, to 358.2 million individuals, according to the Global Burden of Diseases, Injuries, and Risk Factors (GBD) 2015 study [2]. In China, the overall prevalence of asthma in 50,991 participants was found to be 4.2%, representing 45.7 million Chinese adults [3]. Patients who experience asthma often have impaired quality of life (QOL), low pulmonary function, descending exercise tolerance and poor symptom control, which may be life-threatening and carry a significant burden for society. While medication is the main approach to asthma, non-pharmacological strategies can be used as supplements. Asthma patients, especially those with one or more risk factors for exacerbations, should consider non-pharmacological strategies and interventions to assist with symptom control and risk reduction.

Pulmonary rehabilitation (PR) is an evidence-based, multidisciplinary, and comprehensive intervention that was designed to improve the physical and psychological condition of patients with chronic respiratory disease [4, 5]. PR includes but is not limited to exercise training, education, and behaviour change [4, 5]. Exercise training is an important part of PR and involves endurance training, interval training, resistance/strength training, and flexibility training, among others. It has been reported to improve asthma symptoms, QOL, exercise capacity, bronchial hyperresponsiveness, exercise-induced bronchoconstriction and cardiopulmonary fitness and to reduce airway inflammation and nocturnal symptoms in patients with asthma [6–12]. However, these studies included both adults and children or focused on airway inflammation alone; furthermore, they are outdated. This meta-analysis aimed to evaluate the effects of exercise-based PR compared to other treatments (standard medical care, educational program, drug treatment, etc.) on QOL, asthma control, pulmonary function and exercise tolerance in adults with chronic persistent or clinically stable asthma.

## Methods

The methods of this review strictly followed the Preferred Reporting Items for Systematic Reviews and Meta-Analyses (PRISMA) statement [13] (see Additional file 1: Table S1).

### Search strategy

The PubMed, Embase, Cochrane Library, Web of Science, International Clinical Trials Registry Platform and ClinicalTrials.gov databases were searched from inception to 31 July 2019 without language restriction. Detailed search strategies were developed for each database related to “asthma” and “pulmonary rehabilitation” (including “exercise training”, “exercise therapy”, “endurance training”, “resistance training”, “muscle stretching exercises”, “upper limb training”, and “interval training”) and “randomized controlled trials (RCTs)”. We also reviewed the references of the included literature and correlated systematic reviews.

### Study selection

The study selection was conducted by two investigators, and any disagreements were resolved through consultation with a third investigator. First, repeated and irrelevant studies were discarded by examining titles and abstracts. Then, the full texts of potentially eligible studies were obtained and reviewed according to inclusion and exclusion criteria.

The inclusion criteria included all of the following: (i) participants with chronic persistent or clinically stable asthma who were greater than 18 years old; (ii) the intervention involved any exercise-based PR techniques, such as endurance training, resistance training, muscle stretching exercises, exercise training, upper limb training, flexibility training and interval training; (iii) at least one of the outcomes measured QOL, asthma control, pulmonary function or exercise tolerance; and (iv) the study design was an RCT.

The exclusion criteria included any of the following: (i) participants with complications of any other pulmonary diseases in addition to asthma; (ii) the literature type was a study protocol; and (iii) the full texts could not be obtained, such as with meeting abstracts or supplements.

### Data extraction

Data extraction was conducted by two investigators, and disagreements were resolved by a third investigator. Information including author information, publication year, study design, region, participants (age, sex and sample size), interventions, comparator and outcomes was extracted. The primary outcome measure was QOL, as measured by the asthma QOL questionnaire (AQLQ) [14]. The secondary outcome measures were as follows: (i) asthma control, as measured by asthma control questionnaire (ACQ) [15, 16] and asthma symptom-free days; (ii) pulmonary function, measured by forced expiratory volume in 1 s (FEV_1_), forced vital capacity (FVC) and peak expiratory flow (PEF); and (iii) exercise tolerance, as measured by the 6-min walk distance (6 MWD) and maximum oxygen uptake (VO_2_ max). When studies provided insufficient data for meta-analysis, we contacted the first author or corresponding author by email to determine whether additional data could be provided to us.

### Risk of bias evaluation

Risk of bias was evaluated by two investigators, with a third investigator acting as an arbiter in the case of inconsistency, using the Cochrane risk of bias tool [17]. Aspects evaluated included random sequence generation, allocation concealment, blinding of participants and personnel, blinding of outcome assessment, incomplete outcome data, selective reporting and other bias. Each study was scored with “low risk of bias”, “unclear risk of bias” or “high risk of bias”.

### Data analysis

The meta-analysis was conducted using RevMan software (version 5.3). Data are presented as the mean and standard deviation (SD). The effect size was estimated by the mean difference (MD) with 95% confidence intervals (CIs) for continuous outcome measures. In the case of different scales (e.g., ACQ-6 and ACQ-7) for the same outcome measure, the standardized MD (SMD) was chosen [17]; a missing SD was calculated if possible. A fixed-effect model was applied if there was no statistically significant heterogeneity; otherwise, a random-effect model was employed [17]. The χ^2^ test with *P* < 0.1 or I^2^ > 50% indicated significant heterogeneity [18]. Additionally, we examined the effects of exercise-based PR on different domains of the QOL scale. If the data could not be assessed by meta-analyses, we summarized them in the text in qualitative ways.

### Evidence quality evaluation

The quality of evidence for primary outcomes was evaluated using GRADEpro (GRADEproGDT, http://www.gradepro.org/) [19]. Factors downgrading the evidence quality (risk of bias, inconsistency, indirectness, imprecision, and publication bias) were rated, and the evidence quality was assessed as “very low”, “low”, “moderate” or “high”.

## Results

### Literature search and study selection

**Fig. 1:**
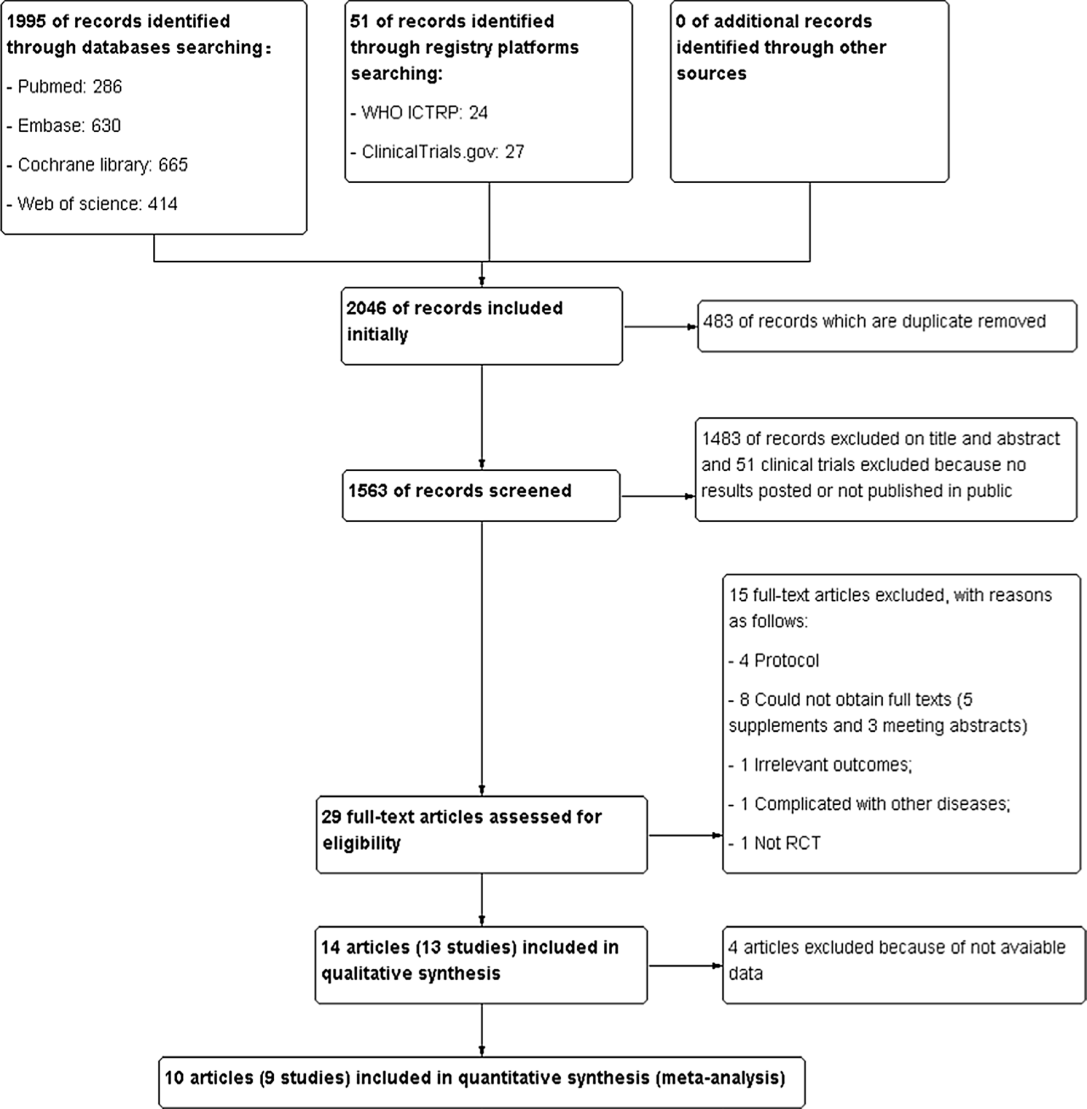
Study flow diagram

A total of 2046 records were identified. Fourteen literatures [20–33] from 13 studies were included in the qualitative synthesis, and 10 literatures [24–33] from 9 studies, including 418 participants, were eventually included in the meta-analysis. The process of study selection is shown in Fig. 1.

### Data extraction and risk of bias evaluation

The basic characteristics extracted from the included literatures are shown in Table 1. The risk of bias evaluation is summarized in Additional file 2: Figures S1, and Additional file 3: Figures S2.

**Table 1:** Basic characteristics of the included studies

Study	Country	Design	Participants	Interventions	Outcomes	Notes
França-Pinto et al. [24]	Brazil	RCT, 2 arms	*Participant status:* Sex(F/M): EG: 17/5; CG: 17/4 Age(years): EG: 40 ± 11; CG: 44 ± 9 *Participants randomly assigned:* 43 participants were randomly assigned Analysed: EG: 22; CG: 21	EG: breathing exercise programme + educational programme + aerobic training programme CG: breathing exercise programme + educational programme Duration of treatments: 3 months	IL-5, IL-6, IL-8, IL-10, MCP-1, IgE, clinical control (asthma symptom-free days, ACQ), AQLQ, induced sputum, exercise capacity (VO_2_ max, maximal workload, pulmonary function (FEV_1_, FEV_1_% predicted)	
Cochrane and Clark [25]	Scotland	RCT, 2 arms	*Participant status:* Sex(F/M): 22/14 Age(years): EG: 27 ± 7; CG: 28 ± 8 *Participants randomly assigned:* 36 participants were randomly assigned Analysed: EG: 18; CG: 18	EG: physical training (aerobic exercises, stretching exercises) + educational sessions CG: educational sessions Duration of treatments: 3 months	FEV_1_, FEV_1_% pre, VO_2_ max, oxygen pulse, body fat, cholesterol, LDL, HDL, max heart rate, VE max, V_T_, RR, V_EO2_, DI max (%)	
Refaat and Gawish [26]	Kuwait	RCT, 2 arms	*Participant status:* Sex(F/M): EG: 21/17; CG: 16/14 Age(years): EG: 35.8 ± 1.7; CG: 38 ± 5.3 *Participants randomly assigned:* 68 participants were randomly assigned Analysed: EG: 38; CG: 30	EG: physical training + standard medical care CG: standard medical care Duration of treatments: 3 months	AQLQ, pulmonary function (FEV_1_, FVC, PEF)	
Toennesen et al. [27]	Denmark	RCT, 4 arms	*Participant status:* Sex(F/M): EG: 22/7; CG: 25/8 Age(years): EG: 43.7 ± 13.9; CG: 40.7 ± 14.7 *Participants randomly assigned:* 62 participants were randomly assigned Analysed: EG: 29; CG: 33	EG: high-intensity interval training CG: diet Duration of treatments: 8 weeks	AQLQ, ACQ, VO_2_ max, FEV_1_%pred, FVC %pred, F_ENO_, serum IL-6, serum hs-CRP, blood eosinophils, sputum eosinophils (%), sputum neutrophils (%)	Data from exercise and diet groups were analysed
Turner et al. [28]	Australia	RCT, 2 arms	*Participant status:* Sex(F/M): EG: 11/8; CG: 8/7 Age(years): EG: 65.3 ± 10.8, CG: 71.0 ± 9.7 *Participants randomly assigned:* 34 participants were randomly assigned Analysed: EG: 19; CG: 15	EG: exercise training + standard medical care CG: standard medical care Duration of treatments: 3 months	AQLQ, ACQ, SF-36, 6 MWD, HADS, peak heart rate, SpO_2_ end test, dyspnoea end test, Quadriceps strength (% of pre), Hand grip strength (% of pre)	
Shaw and Shaw [29]	South Africa	RCT, 4 arms	*Participant status:* Sex(F/M): EG: 8/14; CG: 8/14 Age(years): EG: 21.95 ± 3.87; CG: 21.90 ± 3.89 *Participants randomly assigned:* 44 participants were randomly assigned Analysed: EG: 22; CG: 22	EG: aerobic exercise CG: normal daily activities Duration of treatments: 8 weeks	FEV_1_, FVC, FEV_1_/FVC, PEF, MVV, IVC, VE, VT, mean chest circumferences at the height of the second intercostal space	Data from aerobic exercise (AE) and nonexercise control (NE) groups were analysed
Cambach et al. [30]	the Netherlands	RCT, 2 arms	*Participant status:* Sex(F/M): EG: 18/4; CG: 14/7 Age(years): EG: 40 ± 10; CG: 53 ± 15 *Participants randomly assigned:* 43 participants were randomly assigned Analysed: EG: 22; CG: 21	EG: PR + drug treatment CG: drug treatment Duration of treatments: 3 months	QOL, exercise tolerance (endurance time, cardiac frequency, 6 MWD)	A crossover design study, data of phase 1(from baseline to 3 months) were analysed
Freitas et al. [31]	Brazil	RCT, 2 arms	*Participant status:* Sex(F/M): EG: 25/1; CG: 25/0 Age(years): EG: 45.9 ± 7.7; CG: 48.5 ± 9.6 *Participants randomly assigned:* 51 participants were randomly assigned Analysed: EG: 26; CG: 25	EG: a weight-loss programme + exercise CG: a weight-loss programme + sham exercise Duration of treatments: 3 months	AQLQ, ACQ, pulmonary function (FEV_1_, FVC, TLC, ERV), strength of muscle, VO_2_ max, work rate	Sham exercise; stretching exercise and breathing exercise that did not affect asthma control
Freitas et al. [32]	Brazil	RCT, 2 arms	*Participant status:* Sex(F/M): EG: 25/1; CG: 25/0 Age(years): EG: 45.9 ± 7.7; CG: 48.5 ± 9.6 *Participants randomly assigned:* 51 participants were randomly assigned Analysed: EG: 26; CG: 25	EG: a weight-loss programme + exercise CG: a weight-loss programme + sham exercise Duration of treatments: 3 months	asthma symptom-free days	Sham exercise; stretching exercise and breathing exercise that did not affect asthma control
Coelho et al. [33]	Brazil	RCT, 2 arms	*Participant status:* Sex(F/M): EG: 18/2; CG: 14/3 Age(years): EG: 45.0 ± 19.0; CG: 47.0 ± 14.0 *Participants randomly assigned:* 37 participants were randomly assigned Analysed: EG: 20; CG: 17	EG: physical activity + usual care CG: usual care Duration of treatments: 3 months	AQLQ, ACQ, HADS, daily steps, 6 MWD	

*F* female, *M* male, *IL-5* interleukin 5, *MCP* monocyte chemoattractant protein, *IgE* immunoglobulin E, *LDL* low-density lipoprotein, *HDL* high-density lipoprotein, *V*_*E*_ minute venFtilation, *V*_*T*_ maximum tidal volume, *RR* maximum respiratory rate, *V*_*EO2*_ ventilatory equivalent for oxygen at maximal exercise, *DI max* dyspnoea index at maximal exercise, *F*_*ENO*_ fractional exhaled nitric oxide, *CRP* C-reactive protein, *HADS* hospital anxiety and depression scale, *SpO*_*2*_ percutaneous oxygen saturation, *MVV* maximal voluntary ventilation, *IVC* inspiratory vital capacity, *TLC* total lung capacity, *ERV* expiratory reserve volume

### Effects of inventions

The results of the meta-analyses are shown in Table 2.

**Table 2:** Meta-analysis of exercise-based PR for asthma

Outcomes		No. of RCTs	No. of participants	Effect estimate (95% CI)	*I*^2^ (%)	*P* value
*Primary outcome measures*						
Quality of life						
	AQLQ (Overall QOL)	4	198	MD 0.39 (0.02, 0.76)	0	0.04
	AQLQ (Activity domain)	4	196	MD 0.58 (0.21, 0.94)	0	0.002
	AQLQ (Symptom domain)	4	196	MD 0.52 (0.19, 0.85)	0	0.002
	AQLQ (Emotion domain)	4	196	MD 0.53 (− 0.03, 1.09)	0	0.06
	AQLQ (Environment domain)	4	196	MD 0.56 (0.00, 1.11)	0	0.05
*Secondary outcome measures*						
Asthma control	ACQ	5	215	SMD − 0.25 (0.51, 0.02)	0	0.07
	Asthma symptom-free days	2	94	MD 3.35 (− 0.21, 6.90)	17	0.07
Pulmonary function	FEV_1_	5	242	MD 0.10 (− 0.08, 0.29)	74	0.28
	FVC	3	163	MD 0.23 (0.08, 0.38)	0	0.003
	PEF	2	112	MD 0.39 (0.21, 0.57)	0	< 0.0001
Exercise tolerance	6 MWD	3	94	MD 34.09 (2.51, 65.66)	0	0.03
	VO_2_ max	3	141	MD 4.45 (3.32, 5.58)	0	< 0.00001

*RCTs* randomized controlled trials, *CI* confidence interval, *AQLQ* asthma quality of life questionnaire, *MD* mean difference, *ACQ* asthma control questionnaire, *SMD* standardized MD, *FEV*_*1*_ forced expiratory volume in 1 s, *FVC* forced vital capacity, *PEF* peak expiratory flow, *6 MWD* 6-min walk distance, *VO*_*2*_* max* maximum oxygen uptake

#### AQLQ

**Fig. 2:**
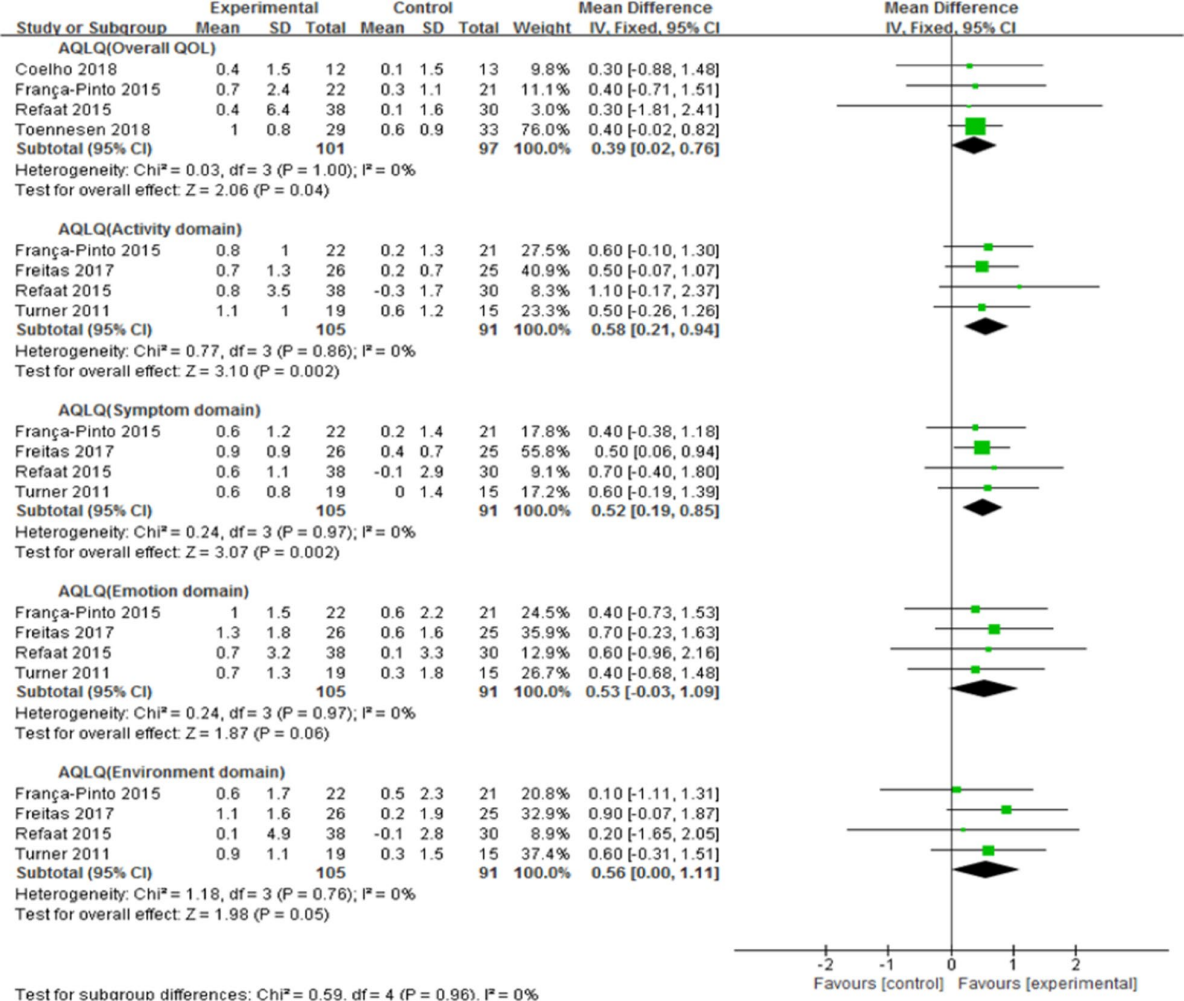
Forest plot of exercise-based PR on AQLQ in patients with asthma. AQLQ: asthma quality of life questionnaire; SD: standard deviation; CI: confidence interval

Six studies [24, 26–28, 31, 33] (283 participants) provided numerical data for the AQLQ and were included in the meta-analysis. Among them, four studies [24, 26, 27, 33] (198 participants) provided the total AQLQ scores; there was no statistical heterogeneity (χ^2^ = 0.03, *P* = 1.00; I^2^ = 0%), and a fixed-effects model was adopted. The pooled results showed that the total AQLQ scores in the experimental group (EG) were significantly improved compared to those in the control group (CG) (MD, 0.39; 95% CI, 0.02 to 0.76; *Z* = 2.06, *P* = 0.04). Four studies [24, 26, 28, 31] (196 participants) provided AQLQ domain scores. For the activity domain, there was no statistical heterogeneity (χ^2^ = 0.77, *P* = 0.86; I^2^ = 0%), and a fixed-effects model was utilized. The activity domain scores in EG improved more than those in CG (MD, 0.58; 95% CI, 0.21 to 0.94; *Z* = 3.10, *P* = 0.002). In the symptom domain, there was no statistical heterogeneity (χ^2^ = 0.24, *P* = 0.97; I^2^ = 0%), and a fixed-effect model was used. The symptom domain scores in EG improved more than those in CG (MD, 0.52; 95% CI, 0.19 to 0.85; *Z* = 3.07, *P* = 0.002). There was no statistical heterogeneity in the emotion domain (χ^2^ = 0.24, *P* = 0.97; I^2^ = 0%), and a fixed-effect model was used. According to pooled data, there was no statistically significant improvement between the two groups (MD, 0.53; 95% CI, -0.03 to 1.09; *Z* = 1.87, *P* = 0.06). Regarding the environment domain, there was no statistical heterogeneity (χ^2^ = 1.18, *P* = 0.76; I^2^ = 0%), and a fixed-effect model was selected. The pooled data showed no statistically significant improvement between the two groups (MD, 0.56; 95% CI, 0.00 to 1.11; *Z* = 1.98, *P* = 0.05) (Fig. 2).

#### ACQ

Six studies [20, 24, 27, 28, 31, 33] investigated the effects of exercise-based PR on asthma control by ACQ. Among them, five studies [24, 27, 28, 31, 33] (215 participants) provided numerical data for ACQ scores and were included in the meta-analysis. There was no statistical heterogeneity (χ^2^ = 0.73, *P* = 0.95; I^2^ = 0%), and a fixed-effect model was applied. The data revealed no statistically significant improvement between the two groups (MD, − 0.25; 95% CI, − 0.51 to 0.02; *Z* = 1.79, *P* = 0.07). One study [20], which was not included in the meta-analysis, reported a mean improvement in asthma control of 0.22 versus 0.73, but the change was not statistically significant between the two groups (see Additional file 4: Figure S3).

### Asthma symptom-free days

Five studies [21–24, 32] investigated the effects of exercise-based PR on asthma symptom-free days; two studies [24, 32] (94 participants) provided numerical data and were included in the meta-analysis. Because there was no statistical heterogeneity (χ^2^ = 1.20, *P* = 0.27; I^2^ = 17%), a fixed-effect model was used. However, there was no statistically significant improvement between the two groups (MD, 3.35; 95% CI, -0.21 to 6.90; *Z* = 1.84, *P* = 0.07). Three studies [21–23] that were not included in the meta-analysis reported a statistically significant improvement (see Additional file 5: Figure S4).

#### FEV_1_

Seven studies [20, 22, 24–26, 29, 31] investigated the effects of exercise-based PR on FEV_1_. Among them, five [24–26, 29, 31] (242 participants) provided numerical data for FEV_1_ and were included in the meta-analysis. There was statistical heterogeneity (χ^2^ = 15.37, *P* = 0.004; I^2^ = 74%); thus, a random-effects model was used. According to the data, there was no statistically significant improvement (MD, 0.10; 95% CI, − 0.08 to 0.29; *Z* = 1.08, *P* = 0.28) between the two groups. Two studies [20, 22] also reported no change in FEV_1_ between the two groups (see Additional file 6: Figure S5).

#### FVC

Four studies [22, 26, 29, 31] investigated the effects of exercise-based PR on FVC. Among them, three studies [26, 29, 31] (163 participants) provided numerical data for FVC and were included in the meta-analysis. There was no statistical heterogeneity (χ^2^ = 0.12, *P* = 0.94; I^2^ = 0%), and a fixed-effect model was used. The data showed greater improvement in EG (MD, 0.23; 95% CI, 0.08 to 0.38; *Z* = 2.94, *P* = 0.003) than in CG. One study [22] reported no change in FVC between the two groups (see Additional file 7: Figure S6).

#### PEF

Two studies [26, 29] (112 participants) provided numerical data for PEF; they were included in the meta-analysis. A fixed-effect model was used due to a lack of statistical heterogeneity (χ^2^ = 0.02, *P* = 0.87; I^2^ = 0%). The data showed a greater effect in EG (MD, 0.39; 95% CI, 0.21 to 0.57; *Z* = 4.32, *P* < 0.0001) than in CG (see Additional file 8: Figure S7).

#### 6 MWD

Three studies [28, 30, 33] (94 participants) provided numerical data for 6 MWD. As there was no statistical heterogeneity (χ^2^ = 1.06, *P* = 0.59; I^2^ = 0%), a fixed-effect model was used. The effect in EG was greater (MD, 34.09; 95% CI, 2.51 to 65.66; *Z* = 2.12, *P* = 0.03) than that in CG (see Additional file 9: Figure S8).

#### VO_2_ max

Five studies [21, 22, 24, 25, 27] investigated the effects of exercise-based PR on VO_2_ max. Among them, three [24, 25, 27] (141 participants) provided numerical data and were included in the meta-analysis. There was no statistical heterogeneity (χ^2^ = 1.50, *P* = 0.47; I^2^ = 0%), and a fixed-effect model was used. The data showed that the effect in EG was superior (MD, 4.45; 95% CI, 3.32 to 5.58; *Z* = 7.74, *P* < 0.00001) to that in CG. Two studies [21, 22] that were not included in the meta-analysis also reported a significant increase in VO_2_ max between the two groups (see Additional file 10: Figure S9).

#### Evidence quality evaluation

The overall AQLQ and every domain were rated as having “moderate quality” due to small sample sizes and wide confidence intervals. The GRADE evidence profile is presented in Table 3.

**Table 3:** Quality of evidence for primary outcomes in patients with asthma

Certainty assessment							No. of patients		Effect		Certainty	Importance
No. of included studies	Study design	Risk of bias	Inconsiste-ncy	Indirectness	Imprecision	Other considerat-ions	Experimental group	Control group	Relative (95% CI)	Absolute (95% CI)		
*Asthma quality of life—AQLQ (overall QOL)*												
4	RCT	Not serious	Not serious	Not serious	Serious^a^	None	101	97	–	MD 0.39 higher (0.02 higher to 0.76 higher)	⨁⨁⨁◯ Moderate	Critical
*Asthma quality of life—AQLQ (activity domain)*												
4	RCT	Not serious	Not serious	Not serious	Serious^a^	None	105	91	–	MD 0.58 higher (0.21 higher to 0.94 higher)	⨁⨁⨁◯ Moderate	Critical
*Asthma quality of life—AQLQ (symptom domain)*												
4	RCT	Not serious	Not serious	Not serious	Serious^a^	None	105	91	–	MD 0.52 higher (0.19 higher to 0.85 higher)	⨁⨁⨁◯ Moderate	Critical
*Asthma quality of life—AQLQ (emotion domain)*												
4	RCT	Not serious	Not serious	Not serious	Serious^a^	None	105	91	–	MD 0.53 higher (0.03 lower to 1.09 higher)	⨁⨁⨁◯ Moderate	Critical
*Asthma quality of life—AQLQ (environment domain)*												
4	RCT	Not serious	Not serious	Not serious	Serious^a^	None	105	91	–	MD 0.56 higher (0 to 1.11 higher)	⨁⨁⨁◯ Moderate	Critical

*CI* confidence interval, *RCT* randomized controlled trial, *AQLQ* asthma quality of life questionnaire, *MD* mean difference

^a^Small sample size and wide confidence interval

## Discussion

PR is a comprehensive intervention designed to improve the physical and psychological condition of people with chronic respiratory disease and to promote long-term adherence to health-enhancing behaviours [34]. In recent years, quite a few studies have confirmed the positive effects of PR on patients with respiratory conditions, such as cystic fibrosis, bronchiectasis, interstitial lung disease, and lung transplantation [35–38]. Exercise training is part of PR, which has been applied widely. This systematic review and meta-analysis summarized the effects of exercise-based PR on adults with chronic persistent or clinically stable asthma, aiming to provide evidence for clinicians and policy makers.

QOL, an important index for characterizing patient populations and evaluating therapeutic interventions, cannot be captured by biological or clinical indicators. The AQLQ is a widely used instrument to evaluate QOL in those with asthma; it consists of an activity domain, symptom domain, emotion domain and environment domain. Our meta-analysis showed that exercise-based PR significantly improved overall QOL as well as each domain, with a consistent trend, but the improvement in the emotion and environment domains was not statistically significant. The *p* value was 0.06 in the emotion domain, near 0.05; it was 0.05 in the environment domain. This may be due to the small sample size; thus, significant improvement was not detected. From another perspective, many people with asthma also have emotional problems, such as anxiety and depression. This is usually related to multiple factors, including older age, lower income, use of oral corticosteroids, patients’ perceived severity of asthma, disability, social support and personality traits [39]. In addition, the environmental domain of AQLQ includes cigarette smoke, dust, weather or air pollution outside, and strong smells or perfumes. Environmental risk factors are challenging in adult-onset asthma and play an important role in asthma or related phenotypes [40]. The dynamic and unique biological responses triggered by allergens and air pollutants have proven difficult to predict and prevent [41]. Thus, exercise-based PR alone may not be effective owing to the complexity of emotional and environmental aspects. RCTs with larger sample sizes are necessary to prove the effects of exercise-based PR on these domains of the AQLQ.

Asthma control levels were evaluated by both the ACQ and asthma symptom-free days. There was no statistically significant decrease (a higher score on the ACQ indicates worse asthma control), with an SMD of − 0.25. A recent study [42] reported that regular exercise improves asthma control in adults, which is opposite to our results. Probable explanations are as follows. First, the intervention time of our included studies referring to the ACQ was no more than three months, while that of the previous study was six months. Second, the measurement instrument of our review was the ACQ, while the instrument used in the previous study was the asthma control test. For asthma symptom-free days, there was no statistically significant decrease, with an MD of 3.35 days. Exercise-based PR in proper time and intensity may improve asthma control.

Pulmonary function tests are applied for diagnosing and monitoring at the patient level and for evaluating population trends in respiratory disease over time [43]. As reported in a previous meta-analysis [9], there was no significant improvement in FEV_1_. In terms of FVC, there was significant improvement in three studies [26, 29, 31]. However, one study [22] reported no change between the two groups, and it was not included in the meta-analysis because data were reported as medians and interquartile ranges. A statistically significant improvement in PEF was detected in our review, in contrast to the meta-analysis conducted by Carson and Ram [8, 10]. Divergent opinions exist regarding the effects of exercise-based PR on the pulmonary function of asthma patients, and this may be due to small sample sizes, different exercise durations and intensities, and asthma severity, among others.

Exercise tolerance was evaluated by 6 MWD and VO_2_ max in our review. EG had a statistically significant improvement by 34.09 m compared with CG. There was a statistically significant improvement in VO_2_ max, with an MD of 4.45, which is in accordance with a previous meta-analysis [6, 8, 9]. These results support the idea that exercise-based PR enhances exercise tolerance.

There were some limitations in our study. First, the sample size was small, leading to imprecision of outcomes. Nonetheless, this is the only systematic review and meta-analysis to date evaluating the effectiveness of exercise-based PR in adults with asthma. Second, there were various forms of exercise-based PR, which made it difficult to evaluate the effect of a single form. Third, the characteristics of interventions made it difficult to implement blinding, leading to potential performance bias. Finally, four studies could not be included in the quantitative analysis because of the original forms of data reported, such as medians or quartiles; thus, the data could not be used fully.

## Conclusions

Exercise-based PR may improve the QOL, exercise tolerance and pulmonary function of adults with asthma and can be considered a supplementary therapy for asthma management. Additionally, asthma control may be enhanced with proper time and intensity of exercise-based PR. RCTs of high quality and large sample sizes are required for further research.

## Supplementary Information

**Additional file 1: Table S1. **The PRISMA Checklist.

**Additional file 2: Figure S1.** Risk of bias evaluation.

**Additional file 3: Figure S2.** Risk of bias evaluation.

**Additional file 4: Figure S3.** Funnel plots of all studies for each secondary outcome measure.

**Additional file 5: Figure S4.** Funnel plots of all studies for each secondary outcome measure.

**Additional file 6: Figure S5.** Funnel plots of all studies for each secondary outcome measure.

**Additional file 7: Figure S6.** Funnel plots of all studies for each secondary outcome measure.

**Additional file 8: Figure S7.** Funnel plots of all studies for each secondary outcome measure.

**Additional file 9: Figure S8.** Funnel plots of all studies for each secondary outcome measure.

**Additional file 10: Figure S9.** Funnel plots of all studies for each secondary outcome measure.

## Data Availability

All data generated or analyzed during the present study are included in this published article and its supplementary information files.

## Abbreviations

PR: Pulmonary rehabilitation
GRADE: Grading of Recommendations, Assessment, Development and Evaluation
RCT: Randomized controlled trial
PRISMA: Preferred Reporting Items for Systematic Reviews and Meta-Analyses
EG: Experimental group
CG: Control group
QOL: Quality of life
SD: Standard deviation
MD: Mean difference
CI: Confidence interval
F: Female
M: Male
IL-5: Interleukin 5
MCP: Monocyte chemoattractant protein
IgE: Immunoglobulin E
ACQ: Asthma control questionnaire
AQLQ: Asthma quality of life questionnaire
VO_2_ max: Maximum oxygen uptake
FEV_1_: Forced expiratory volume in 1 s
LDL: Low density lipoprotein
HDL: High density lipoprotein
VE: Minute ventilation
VT: Maximum tidal volume
RR: Maximum respiratory rate
VEO_2_: Ventilatory equivalent for oxygen at maximal exercise
DI max: Dyspnea index at maximal exercise
FVC: Forced vital capacity
PEF: Peak expiratory flow
FENO: Fractional exhaled nitric oxide
CRP: C-reactive protein
6 MWD: 6-Min walk distance
HADS: Hospital anxiety and depression scale
SpO_2_: Percutaneous oxygen saturation
MVV: Maximal voluntary ventilation
IVC: Inspiratory vital capacity
TLC: Total lung capacity
ERV: Expiratory reserve volume
