# Supplementary material for: Effects of exercise-based pulmonary rehabilitation on adults with asthma: a systematic review and meta-analysis
Source: Respir Res. 2021 Jan 30;22:33. doi: 10.1186/s12931-021-01627-w (PMC7847170; doi:10.1186/s12931-021-01627-w)
Supplement: Supplementary file 2 — Additional file 2: Figure S1. Risk of bias evaluation. [file 12931_2021_1627_MOESM2_ESM.pdf]

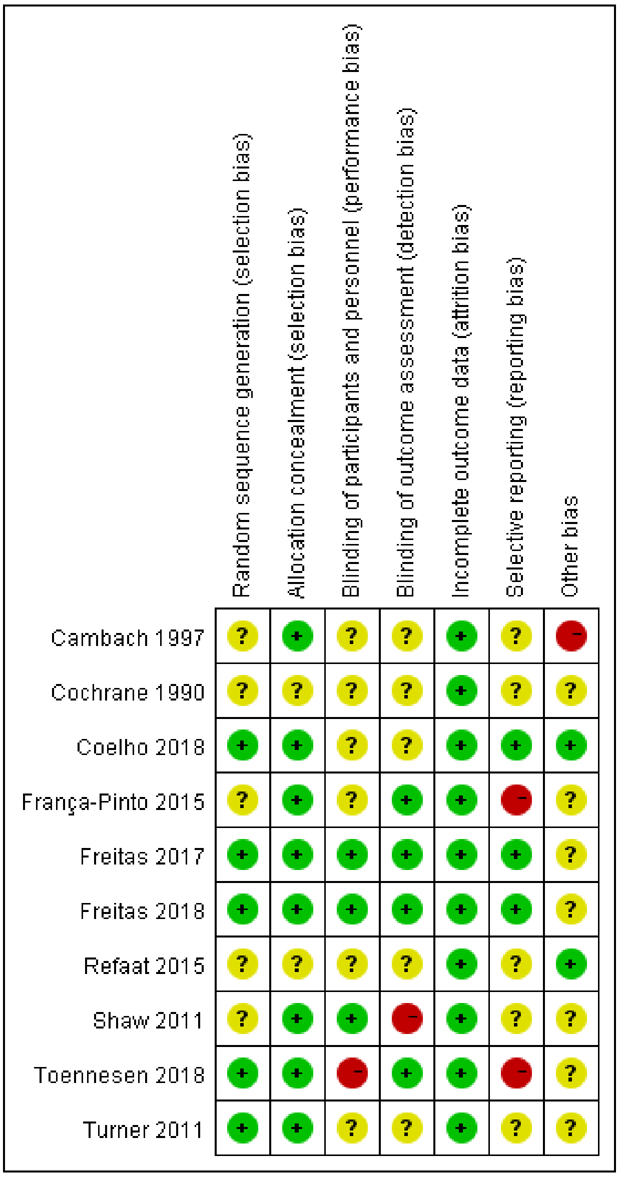

Figure S1 Risk of bias summary: review authors' judgements about each risk of bias item for each included study.
