# Supplementary material for: Effects of exercise-based pulmonary rehabilitation on adults with asthma: a systematic review and meta-analysis
Source: Respir Res. 2021 Jan 30;22:33. doi: 10.1186/s12931-021-01627-w (PMC7847170; doi:10.1186/s12931-021-01627-w)
Supplement: Supplementary file 4 — Additional file 4: Figure S3. Funnel plots of all studies for each secondary outcome measure. [file 12931_2021_1627_MOESM4_ESM.pdf]

Study or Subgroup	Experimental Mean	Experimental SD	Experimental Total	Control Mean	Control SD	Control Total	Weight	Std. Mean Difference IV, Fixed, 95% CI
Coelho 2018	-0.2	1.3	12	0.1	1	13	11.7%	-0.25 [-1.04, 0.54]
França-Pinto 2015	-0.2	0.8	22	-0.1	2.9	21	20.3%	-0.05 [-0.64, 0.55]
Freitas 2017	-0.9	1.5	26	-0.3	1.5	25	23.5%	-0.39 [-0.95, 0.16]
Toennesen 2018	-0.9	0.7	29	-0.7	0.7	33	28.8%	-0.28 [-0.78, 0.22]
Turner 2011	-0.1	0.8	19	0.1	1.1	15	15.7%	-0.21 [-0.89, 0.47]
**Total (95% CI)**			**108**			**107**	**100.0%**	**-0.25 [-0.51, 0.02]**

Heterogeneity: Chi² = 0.73, df = 4 (P = 0.95); I² = 0%
Test for overall effect: Z = 1.79 (P = 0.07)

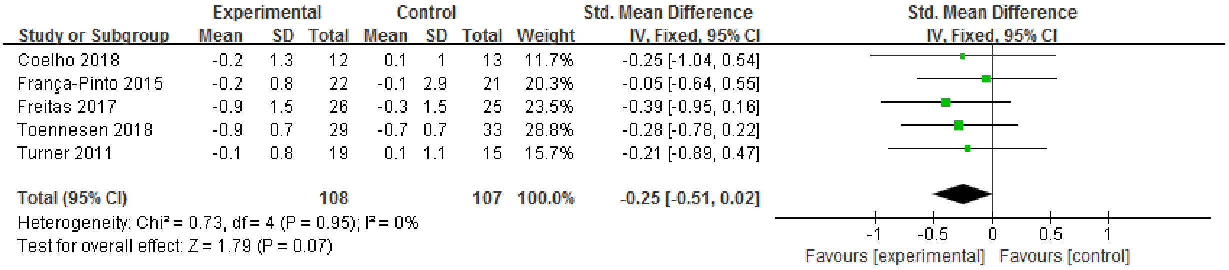

Figure S3 Forest plots of exercise-based PR on ACQ in patients with asthma. ACQ: Asthma control questionnaire; SD: standard deviation; CI: confidence interval
