# Supplementary material for: Effects of exercise-based pulmonary rehabilitation on adults with asthma: a systematic review and meta-analysis
Source: Respir Res. 2021 Jan 30;22:33. doi: 10.1186/s12931-021-01627-w (PMC7847170; doi:10.1186/s12931-021-01627-w)
Supplement: Supplementary file 5 — Additional file 5: Figure S4. Funnel plots of all studies for each secondary outcome measure. [file 12931_2021_1627_MOESM5_ESM.pdf]

Study or Subgroup	Experimental Mean	Experimental SD	Experimental Total	Control Mean	Control SD	Control Total	Weight	Mean Difference IV, Fixed, 95% CI
França-Pinto 2015	4.3	9.2	22	2.5	5.5	21	62.3%	1.80 [-2.71, 6.31]
Freitas 2018	14.5	9.6	26	8.6	11.4	25	37.7%	5.90 [0.10, 11.70]
**Total (95% CI)**			**48**			**46**	**100.0%**	**3.35 [-0.21, 6.90]**

Heterogeneity: Chi² = 1.20, df = 1 (P = 0.27); I² = 17%
Test for overall effect: Z = 1.84 (P = 0.07)

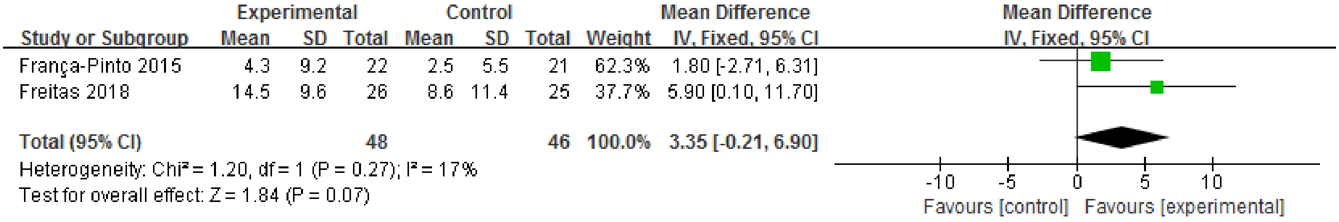

Figure S4 Forest plot of exercise-based PR on asthma symptom-free days in patients with asthma.

SD: standard deviation; CI: confidence interval
