# Supplementary material for: Effects of exercise-based pulmonary rehabilitation on adults with asthma: a systematic review and meta-analysis
Source: Respir Res. 2021 Jan 30;22:33. doi: 10.1186/s12931-021-01627-w (PMC7847170; doi:10.1186/s12931-021-01627-w)
Supplement: Supplementary file 6 — Additional file 6: Figure S5. Funnel plots of all studies for each secondary outcome measure. [file 12931_2021_1627_MOESM6_ESM.pdf]

	Experimental			Control				Mean Difference
Study or Subgroup	Mean	SD	Total	Mean	SD	Total	Weight	IV, Random, 95% CI
Cochrane 1990	0.39	0.69	18	0.19	0.74	18	10.3%	0.20 [-0.27, 0.67]
França-Pinto 2015	0	0.2	22	0.1	0.3	21	25.1%	-0.10 [-0.25, 0.05]
Freitas 2017	0.05	0.3	26	0.06	0.1	25	26.8%	-0.01 [-0.13, 0.11]
Refaat 2015	0.36	0.48	38	-0.01	0.36	30	22.4%	0.37 [0.17, 0.57]
Shaw 2011	0.25	0.6	22	0.08	0.52	22	15.3%	0.17 [-0.16, 0.50]
**Total (95% CI)**			**126**			**116**	**100.0%**	**0.10 [-0.08, 0.29]**

Heterogeneity: $Tau^2 = 0.03$; $Chi^2 = 15.37$, $df = 4$ ($P = 0.004$); $I^2 = 74\%$

Test for overall effect: $Z = 1.08$ ($P = 0.28$)

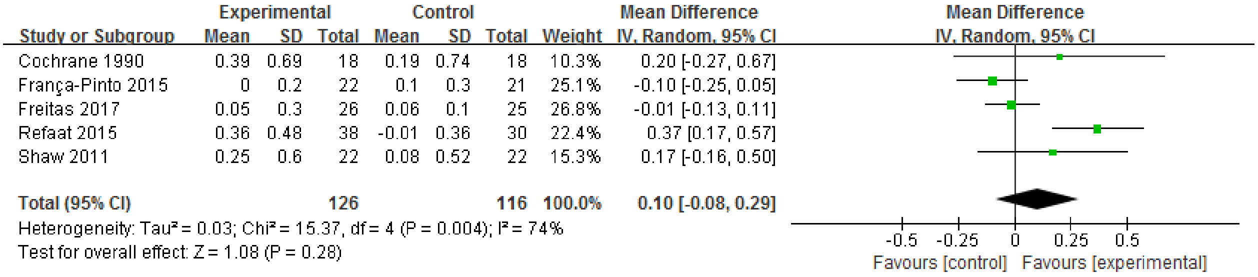

Figure S5 Forest plot of exercise-based PR on FEV_1 in patients with asthma. FEV_1: forced expiratory volume in 1 second; SD: standard deviation; CI: confidence interval
