# Supplementary material for: Effects of exercise-based pulmonary rehabilitation on adults with asthma: a systematic review and meta-analysis
Source: Respir Res. 2021 Jan 30;22:33. doi: 10.1186/s12931-021-01627-w (PMC7847170; doi:10.1186/s12931-021-01627-w)
Supplement: Supplementary file 7 — Additional file 7: Figure S6. Funnel plots of all studies for each secondary outcome measure. [file 12931_2021_1627_MOESM7_ESM.pdf]

Study or Subgroup	Experimental Mean	Experimental SD	Experimental Total	Control Mean	Control SD	Control Total	Weight	Mean Difference IV, Fixed, 95% CI
Freitas 2017	0.2	0.4	26	0	0.4	25	47.1%	0.20 [-0.02, 0.42]
Refaat 2015	0.32	0.51	38	0.06	0.55	30	34.9%	0.26 [0.00, 0.52]
Shaw 2011	0.34	0.63	22	0.11	0.57	22	18.0%	0.23 [-0.13, 0.59]
**Total (95% CI)**			**86**			**77**	**100.0%**	**0.23 [0.08, 0.38]**

Heterogeneity: Chi² = 0.12, df = 2 (P = 0.94); I² = 0%

Test for overall effect: Z = 2.94 (P = 0.003)

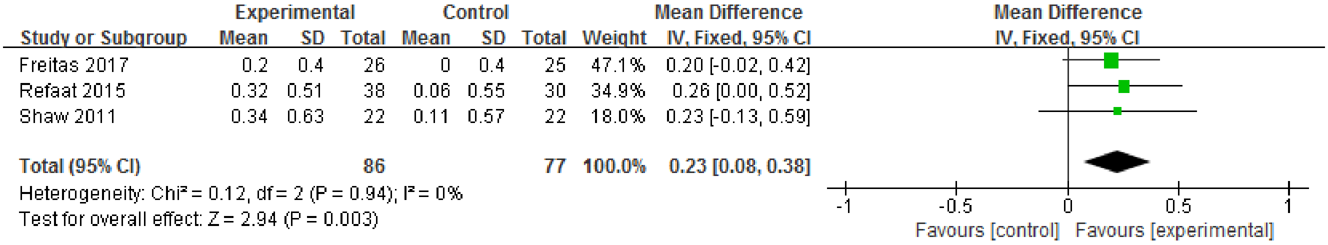

Figure S6 Forest plot of exercise-based PR on FVC in patients with asthma. FVC: forced vital capacity; SD: standard deviation; CI: confidence interval
