# Supplementary material for: Effects of exercise-based pulmonary rehabilitation on adults with asthma: a systematic review and meta-analysis
Source: Respir Res. 2021 Jan 30;22:33. doi: 10.1186/s12931-021-01627-w (PMC7847170; doi:10.1186/s12931-021-01627-w)
Supplement: Supplementary file 8 — Additional file 8: Figure S7. Funnel plots of all studies for each secondary outcome measure. [file 12931_2021_1627_MOESM8_ESM.pdf]

Study or Subgroup	Experimental Mean	Experimental SD	Experimental Total	Control Mean	Control SD	Control Total	Weight	Mean Difference IV, Fixed, 95% CI
Refaat 2015	0.27	0.45	38	-0.12	0.31	30	96.7%	0.39 [0.21, 0.57]
Shaw 2011	0.42	1.46	22	-0.05	1.83	22	3.3%	0.47 [-0.51, 1.45]
**Total (95% CI)**			**60**			**52**	**100.0%**	**0.39 [0.21, 0.57]**

Heterogeneity: Chi² = 0.02, df = 1 (P = 0.87); I² = 0%

Test for overall effect: Z = 4.32 (P < 0.0001)

Mean Difference
IV, Fixed, 95% CI

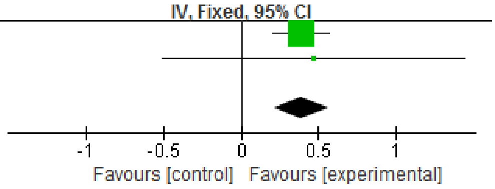

Figure S7 Forest plot of exercise-based PR on PEF in patients with asthma. PEF: peak expiratory flow; SD: standard deviation; CI: confidence interval
