# Supplementary material for: Effects of exercise-based pulmonary rehabilitation on adults with asthma: a systematic review and meta-analysis
Source: Respir Res. 2021 Jan 30;22:33. doi: 10.1186/s12931-021-01627-w (PMC7847170; doi:10.1186/s12931-021-01627-w)
Supplement: Supplementary file 9 — Additional file 9: Figure S8. Funnel plots of all studies for each secondary outcome measure. [file 12931_2021_1627_MOESM9_ESM.pdf]

	Experimental			Control				Mean Difference
Study or Subgroup	Mean	SD	Total	Mean	SD	Total	Weight	IV, Fixed, 95% CI
Cambach 1997	63	89	18	8	63	17	38.5%	55.00 [4.13, 105.87]
Coelho 2018	14.2	44	12	-7.2	76	13	42.9%	21.40 [-26.83, 69.63]
Turner 2011	33	95.8	19	13	116.9	15	18.6%	20.00 [-53.18, 93.18]
**Total (95% CI)**			**49**			**45**	**100.0%**	**34.09 [2.51, 65.66]**

Heterogeneity: Chi² = 1.06, df = 2 (P = 0.59); I² = 0%

Test for overall effect: Z = 2.12 (P = 0.03)

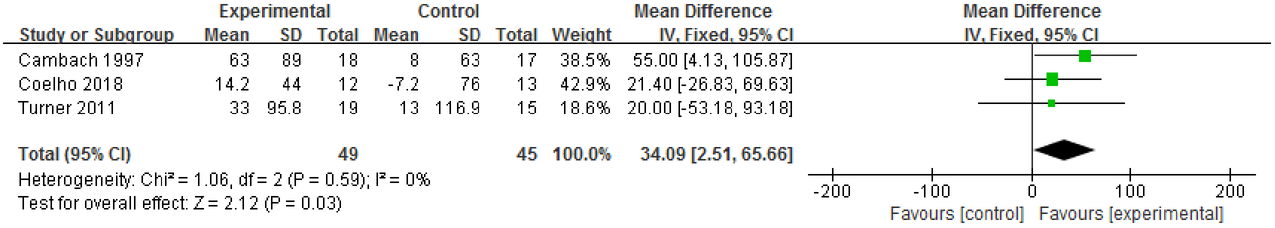

Figure S8 Forest plot of exercise-based PR on 6 MWD in patients with asthma. 6 MWD: 6-minute walk distance; SD: standard deviation; CI: confidence interval
