# Supplementary material for: Effects of exercise-based pulmonary rehabilitation on adults with asthma: a systematic review and meta-analysis
Source: Respir Res. 2021 Jan 30;22:33. doi: 10.1186/s12931-021-01627-w (PMC7847170; doi:10.1186/s12931-021-01627-w)
Supplement: Supplementary file 10 — Additional file 10: Figure S9. Funnel plots of all studies for each secondary outcome measure. [file 12931_2021_1627_MOESM10_ESM.pdf]

Study or Subgroup	Experimental Mean	Experimental SD	Experimental Total	Control Mean	Control SD	Control Total	Weight	Mean Difference IV, Fixed, 95% CI
Cochrane 1990	5.4	5.5	18	-0.9	6.3	18	8.5%	6.30 [2.44, 10.16]
França-Pinto 2015	1	3.3	22	-2.4	5.2	21	18.5%	3.40 [0.78, 6.02]
Toennesen 2018	5.3	2.5	29	0.8	2.8	33	73.0%	4.50 [3.18, 5.82]
**Total (95% CI)**			**69**			**72**	**100.0%**	**4.45 [3.32, 5.58]**

Heterogeneity: Chi² = 1.50, df = 2 (P = 0.47); I² = 0%
Test for overall effect: Z = 7.74 (P < 0.00001)

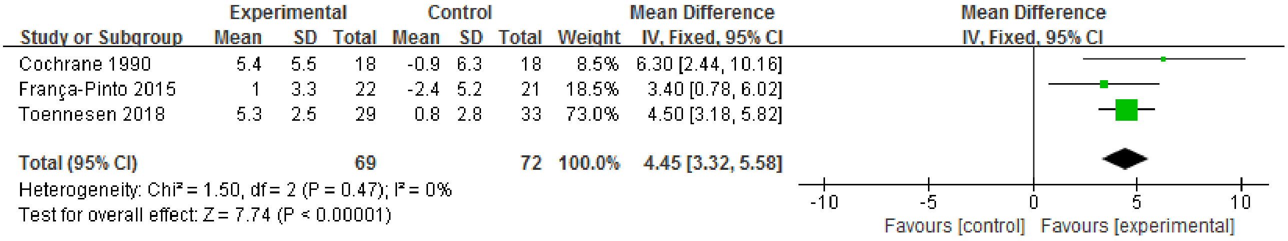

Figure S9 Forest plot of exercise-based PR on VO_2 max in patients with asthma. VO_2 max: maximum oxygen uptake; SD: standard deviation; CI: confidence interval
